# Updated Insights into the Molecular Pathophysiology of Olfactory Neuroblastoma Using Multi-Omics Analysis

**DOI:** 10.3390/jpm15070309

**Published:** 2025-07-13

**Authors:** Enes Demir, Deondra Montgomery, Varun Naravetla, Michael Karsy

**Affiliations:** 1 School of Medicine, Eskisehir Osmangazi University, Eskişehir 26040, Türkiye; enesdemir2002@hotmail.com; 2 College of Human Medicine, Michigan State University, East Lansing, MI 48824, USA; montg353@msu.edu; 3 School of Medicine, University of Michigan, Ann Arbor, MI 48109, USA; vreddyn@umich.edu; 4 Department of Neurosurgery, University of Michigan, Ann Arbor, MI 48109, USA

**Keywords:** esthesioneuroblastoma, olfactory neuroblastoma, genetic alterations, molecular pathways, therapy, molecular markers

## Abstract

**Background/Objectives**: Olfactory neuroblastoma (ONB), also known as esthesioneuroblastoma, is a rare neuroectodermal malignancy of the nasal cavity characterized by aggressive local invasion and variable metastatic potential, with diverse clinical behavior, often presenting at advanced stages. ONB poses challenges for targeted therapeutic strategies, despite advances in surgical and multimodal treatment strategies, because of the rarity of this disease and the limited understanding of its molecular pathophysiology. **Methods**: A comprehensive review of genomic, multi-omic, and molecular studies was performed to integrate known targeted sites in ONB with the current understanding of its pathophysiology. **Results**: Recent genetic and molecular studies have identified significant epigenetic and signaling pathway alterations that are critical in pathogenesis and treatment resistance and may serve as potential therapeutic targets. Additionally, novel discovered immunohistochemical and transcriptomic markers, such as *IDH2*, *NEUROD1*, and *OTX2*, offer improved diagnostic specificity and prognostication. Multi-genomic platforms (i.e., multi-omics), involving the combined integration of transcriptomics, epigenetics, and proteomics findings, have led to several recent insights, including the subclassification of neural and basal genomic subtypes, the identification of key driver mutations, and new insights into disease development. This review synthesizes current knowledge on the molecular landscape of ONB, including its tumor origin, immune microenvironment, genetic alterations, and key molecular pathways involved in its pathogenesis. **Conclusions**: Future research may benefit from integrating these findings into precision medicine approaches, enabling earlier diagnosis and more accurate prognosis.

## 1. Introduction

Olfactory neuroblastoma (ONB), known as esthesioneuroblastoma, is an uncommon, locally aggressive malignancy derived from olfactory epithelial tissue in the upper part of the nasal cavity. First described in 1924 by Berger and Richard [1], ONB is a malignant neuroectodermal tumor, originally thought to originate from olfactory receptor cells located in the superomedial aspect of the nasal cavity and carry neuroendocrine features [2,3,4]. More recent studies have gained further insight into the origin of this tumor [5,6,7].

ONB accounts for approximately 3–5% of sinonasal malignancies, with an incidence of 0.4–1:1,000,000 individuals [8,9,10,11]; it shows no sex or race differences [12] and generally presents at an advanced stage with metastases in developing countries [13]. ONB presents with a peak between the fourth and sixth decades of life [10,11], although ONB has traditionally been considered to present in a bimodal age distribution. No specific risk factors have been identified for ONB [10,14].

The early diagnosis of ONB is difficult because of the non-specific nature of early symptoms [8,15]. Distant metastasis often involves the neck, lungs, and bones, and, rarely, the liver and mediastinum, occurring in around 12% of patients even a decade after diagnosis [8,16]. Metastasis to cervical lymph nodes is not common at presentation but it may occur in between 5 and 25% of cases depending on the stage [8,16].

The four-tiered Hyams grading system classifies ONB into four grades (I–IV), in which prognosis worsens with higher grades, based on histologic features (architecture, pleomorphism, etc.), mitotic activity, and necrosis. It remains the gold standard for histopathologic classification. The most widely accepted clinical staging system for ONB is the Kadish system, which classifies olfactory neuroblastoma as stages A (nasal cavity), B (nasal cavity + sinuses), C (extension beyond sinuses), and D (nodal/distant metastasis), with prognosis worsening by stage [4]. Multimodal treatment involves a combination of surgery and radiotherapy, which seems to be the optimal approach for local advanced resectable cancers [8,11]. For advanced disease, treatment typically involves a multidisciplinary approach, incorporating surgery, radiation, and systemic therapy as essential elements [11,15].

The precise pathophysiology of ONB remains unclear and challenging to understand given the rarity of the disease and the limited number of in vitro or animal models. Recent advances in molecular profiling, including genetic and epigenetic research, have provided insights into the mechanisms underlying ONB pathogenesis and novel aspects of its pathogenesis, prognosis, and potential therapeutic targets, necessitating a comprehensive review of its molecular insights. This review brings together current insights into the molecular basis of ONB, emphasizing the critical role of interdisciplinary collaboration in filling research gaps and improving patient outcomes.

## 2. Pathogenesis

### 2.1. Neural Crest Origin

ONB was initially thought to develop from specialized neuroepithelial olfactory sensory neurons (OSNs). In 1995, Carney et al. performed the first molecular genetic analysis of ONB to determine its origin from immature neural crest cells of the olfactory epithelium [17]. ONB expresses the Drosophila achaete-scute gene (*hASH1*) but not olfactory marker protein (OMP) mRNA. *hASH1* is known to be involved in immature olfactory neuronal development, whereas OMP is a marker of mature cells. In a more recent immunohistochemical study, Matayoshi and Otaki found that ONB expresses the olfactory sensory transduction proteins Gαolf, ACIII, and cyclic nucleotide-gated channel-2 (*CNGCA2*), which are known to be specifically expressed in OSNs. They also identified positive staining of anti-Gαolf, anti-ACIII, and anti-CNGCA2 antibodies. These results support an undifferentiated cell state for ONB development. The ONB-associated expression of the three transduction proteins, along with *hASH1* expression, strongly suggests that ONB and OSNs share the same cellular lineage and arise ultimately from the neuroepithelium [6].

Zunitch et al. provided further compelling evidence linking ONB to the malignant transformation of neuronal progenitor cells in the olfactory epithelium—specifically, GBCs [7]. The authors systematically mapped the gene expression profiles of ONB to normal nasal epithelial cell types by leveraging an integrated single-cell RNA-sequencing (scRNA-seq) atlas from human and murine olfactory mucosa. Their analysis revealed that ONB shares a high degree of transcriptomic similarity with the neuronal progenitor (npGBC) population. The study identified two transcriptomic modules involved in GBC differentiation. One transcriptome was linked to stem-like multipotent states (msGBC) and another was associated with neuronal maturation. ONB samples predominantly expressed the msGBC-to-npGBC transition module, while the subsequent olfactory sensory neuron differentiation module was largely absent, indicating developmental arrest in an npGBC-like state. Recent genetically engineered mouse models using *Rb1+/Trp53+/Myc+* also identified the GBC population as a putative cell of origin, demonstrating multipotent potential and the potential to generate the rest of the ONB cell population [5].

### 2.2. Tumor Microenvironment

**Figure 1:**
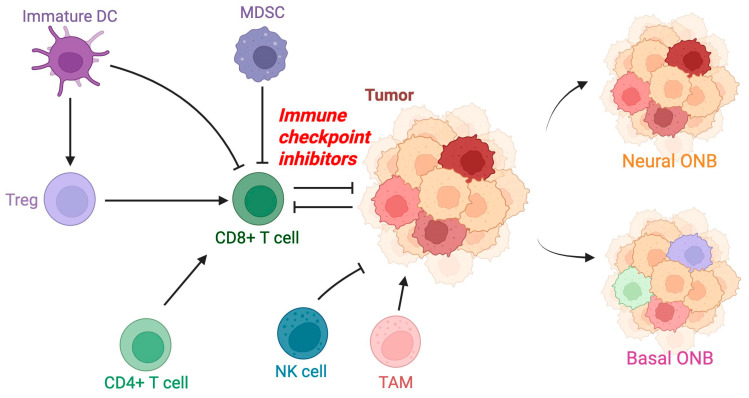
Tumor microenvironment of olfactory neuroblastoma. Some important factors involved in the regulation of the tumor microenvironment of olfactory neuroblastoma are shown based on the available supportive evidence. Targeted therapy with immunomodulators has been supported as a potential treatment path. Important genomic subclasses demonstrate distinct genomic distinctions. Basal ONB shows greater infiltration of tumor-infiltrating lymphocytes compared with neural ONB. DC: dendritic cell; MDSC: myeloid-derived suppressor cell; NK: natural killer; TAM: tumor-associated macrophage.

The tumor microenvironment (TME) plays a critical role in the prognosis of ONB, immune evasion, and the response to treatment, including immunotherapeutic strategies (Figure 1). While low-grade ONB often responds well to surgical resection and adjuvant radiotherapy, high-grade and recurrent ONB exhibit poor outcomes, necessitating a deeper understanding of its tumor microenvironment (TME) to develop more effective therapeutic strategies [18]. Recent studies utilizing multispectral immunofluorescence and RNA-based spatial transcriptomics have provided valuable insights into the composition and functional dynamics of the ONB TME. Understanding these complex interactions is essential for developing novel immunotherapeutic strategies aimed at improving prognosis and survival in patients with high-grade or recurrent ONB [18].

### 2.3. Immune Cell Infiltration

The ONB TME comprises various immune cell populations, including T cells, myeloid-derived suppressor cells (MDSCs), and natural killer (NK) cells [18,19]. A hallmark of the ONB TME is the significant presence of CD8+ cytotoxic T cells, CD4+ helper T cells, and regulatory T cells (Tregs). Notably, CD8+ T cells, which play a key role in tumor cell cytotoxicity, are predominantly located in the stromal regions rather than within the tumor nests, suggesting a mechanism of immune exclusion. Furthermore, programmed cell death protein-1 (PD-1)-expressing T cells are present in the ONB TME, implicating the potential efficacy of immune checkpoint blockade therapies targeting the PD-1/PD-L1 axis in ONB’s immunosuppressive landscape.

### 2.4. Myeloid-Derived Suppressor Cells and Immune Suppression

Myeloid-derived suppressor cells (MDSCs), particularly polymorphonuclear MDSCs (PMN-MDSCs) and monocytic MDSCs (M-MDSCs), are prominent components of the ONB TME and contribute to immune suppression [20]. These cells are more frequently localized within the stromal compartments and are associated with higher grades (Kadish C/D) and poor prognostic features. The presence of MDSCs correlates with increased expression of interleukin-8 (IL-8), a known driver of neutrophil and MDSC recruitment, further reinforcing the immunosuppressive milieu within ONB tumors. Additionally, tumor-associated macrophages (TAMs), marked by CD68 expression, are abundant in the ONB TME and likely contribute to an immunosuppressive environment.

### 2.5. Natural Killer Cells and Immune Evasion

Natural killer (NK) cells are notably sparse within the ONB TME despite their established role in innate tumor surveillance [21]. This scarcity may be attributed to a combination of factors, including the limited tumor cell expression of ligands necessary for NK cell activation and the suppressive influence of the surrounding immune microenvironment. The deficiency of activated NK cells within ONB tumors underscores a potential barrier to immune-mediated tumor clearance and suggests a targetable axis for therapeutic intervention.

### 2.6. Major Histocompatibility Complex Downregulation and Chemokine Signaling

One of the most striking findings in high-grade ONB is the significant downregulation of major histocompatibility complex class I (MHC-I) molecules, which are crucial for antigen presentation to CD8+ T cells. This cold immune environment prevents tumor recognition by cytotoxic T lymphocytes, representing a key mechanism of immune evasion in ONB. MHC-II, which is involved in antigen presentation to CD4+ T cells, is also expressed at low levels in ONB tumor cells.

Chemokines have a pivotal role in the recruitment of immune cells to the tumor site. ONB tumors with higher expression of CXCL9 and CXCL10, chemokines that facilitate CD8+ T cell infiltration, demonstrate an increased immune cell presence within the tumor parenchyma. Conversely, IL-8, which recruits MDSCs and neutrophils, is elevated in the ONB stroma, further reinforcing the immunosuppressive TME. These findings highlight potential targets for immunotherapeutic intervention, as modulating chemokine expression could enhance immune cell infiltration and antitumor responses [22].

## 3. Molecular Factors and Structures

**Figure 2:**
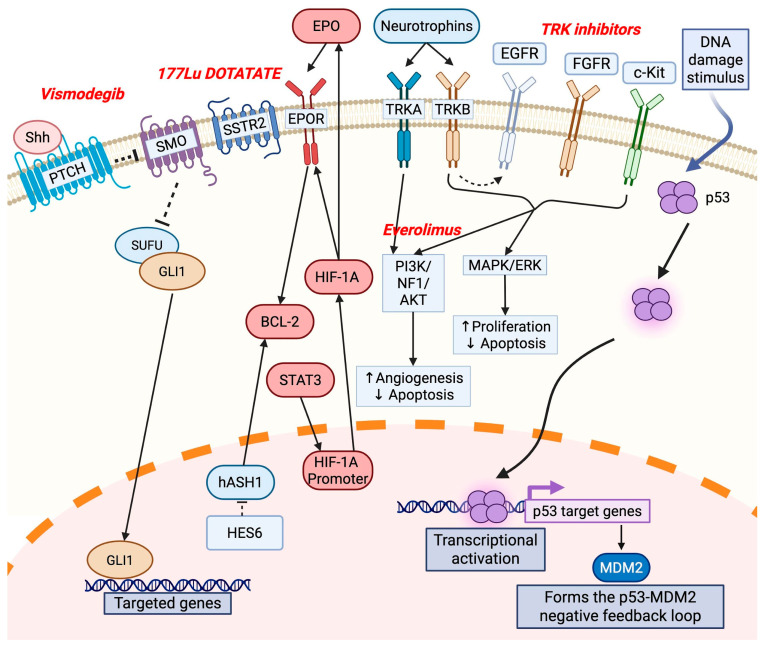
Signaling pathogenesis of olfactory neuroblastoma. Some important signaling factors involved in tumor pathogenesis are shown in olfactory neuroblastoma. Therapeutic targets with vismodegib, 177Lu-Dotatate, tyrosine kinase inhibitors (e.g., sunitinib, pazopanib, imatinib), and everolimus have been investigated. EGFR: epidermal growth factor receptor; FGFR: fibroblast growth factor receptor; HIF-1A: hypoxia-inducible factor 1 alpha; MAPK: mitogen-activated protein kinase; NF1: neurofibromin 1; PI3K: phosphoinositide 3-kinase; PTCH: patched receptor; Shh: sonic hedgehog; SMO: smoothened receptor; SSTR2: somatostatin receptor 2; TRKA: neurotrophic tyrosine receptor kinase.

Studies investigating the molecular pathways involved in ONB pathogenesis in detail are limited. The studies that are available show that the dysregulation of specific markers contributes to ONB pathogenesis (Table 1, Figure 2).

Carney et al. evaluated hASH1 in ONB [44]. RNA interference studies revealed that hASH1 inhibition leads to cell cycle arrest, so its overexpression may act as a trigger for cancer formation from olfactory epithelial cells [45]. Furthermore, the expression of hASH1 is downregulated via the Notch-dependent pathway, pointing to this being a key molecular pathway in the pathogenesis of ONB [44].

The PI3K/AKT and MAPK/ERK pathways have also been implicated in ONB pathogenesis. TrkA and TrkB are neurotrophin receptors that are strongly expressed in almost all ONB cases [32]. Neurotrophins are nerve growth factors that are important in the growth, differentiation, and maintenance of neuronal cell populations [46]. TrkB overexpression participates in tumorigenesis through ERK and Akt pathway activation, which enhances the maintenance of brain tumor-initiating cells (BTICs) [47]. TrkA was not found to be expressed in the BTICs analyzed in this study, pointing to its antiangiogenic effects and association with a favorable prognosis [47,48].

The sonic hedgehog (Shh) signaling pathway plays a significant role in the development and progression of ONB. Mao et al. demonstrated that key components of the Shh pathway—including Patched1, Gli1, and Gli2—are frequently expressed in ONB tumor samples but are absent in normal olfactory epithelial tissue, indicating the pathway’s potential involvement in ONB pathogenesis [49]. An immunohistochemical analysis demonstrated the expression of Patched1, Gli1, and Gli2 in 70%, 70%, and 65% of human ONB cases, respectively, with Patched1 levels inversely and Gli1 levels positively correlating with the tumor severity based on Kadish staging and Hyams grading. Inhibition of the Shh pathway using cyclopamine, a selective transmembrane protein smoothened (SMO) antagonist, suppressed ONB cell proliferation, induced G0/G1 cell cycle arrest, and increased apoptosis in two ONB cell lines (JFEN and TC-268). Cyclopamine treatment led to the downregulation of Patched1, Gli1, and the cell cycle regulator cyclin D1, with the upregulation of the cyclin-dependent kinase inhibitor p21. These effects were partially or completely reversed by exogenous Shh, reinforcing the specificity of this pathway’s role in regulating ONB cell growth and survival. ONB cells were shown to express Shh endogenously, suggesting that the autocrine activation of this pathway may sustain tumor proliferation [49].

bFGF, a specific member of the fibroblast growth factor (FGF) family, has been shown to induce differentiation and apoptosis in ONB cells, highlighting its dual role in modulating tumor cell behavior. While the specific role of bFGF in ONB remains less explored, Nibu et al. investigated the effects of bFGF on ONB using the human JFEN ONB cell line and a xenograft mouse model [50]. Their findings revealed that bFGF induced differentiation and apoptosis in ONB cells, in contrast to its proliferative role in many other tumors. The treatment of JFEN cells with bFGF led to the upregulation of FGFR1, its high-affinity receptor, suggesting an autocrine or paracrine feedback loop. FGFR1 expression was observed in all three major isoforms in JFEN cells, and it was also strongly expressed in the human olfactory epithelium but not in the adjacent respiratory epithelium, supporting the neuroepithelial origin of ONB. bFGF treatment triggered the expression of AML1 (also known as CBFA2), a transcription factor associated with olfactory neuroepithelial differentiation, and TrkA, a nerve growth factor receptor typically expressed in olfactory supporting cells. These findings suggest that bFGF drives ONB cells toward a supporting cell lineage rather than neuronal maturation. In contrast, no induction of TrkB or TrkC—markers of neuronal differentiation—was detected, further supporting this hypothesis [50]. Targeting the bFGF signaling axis in ONB may hold therapeutic promise.

A comprehensive molecular profiling study conducted by Lazo de la Vega et al. identified recurrent amplifications of the *FGFR3* gene in a significant subset of ONB cases [3]. High-level FGFR3 amplification was observed in 5 out of 18 evaluable tumors (28%) in their analysis of 20 ONB tumors. RNA sequencing confirmed that FGFR3 amplification was associated with markedly increased gene expression. *FGFR3* alterations in ONB primarily arose via copy number gains, which were sufficient to induce overexpression. ONB samples lacking *FGFR3* amplification showed significantly lower expression, reinforcing the biological relevance of amplification as a mechanism of activation and demonstrating the potential of *FGFR3* as an oncogenic driver [3]. FGFR-targeted therapies, such as multi-kinase inhibitors, may enable the guidance of targeted treatment modalities.

Angiogenesis is a key component of tumorigenesis. In ONB, the HIF-1a/Epo/EpoR/Bcl-2 system contributes to this process [29]. In this study, the investigators reported the strong spatial distribution of HIF-1α and Epo immunoreactivity in the majority of cases, suggesting that Epo expression in ONB is controlled by HIF-1α [49]. Additionally, they found a significant positive correlation between the expression levels of HIF-1α and Bcl-2 and the microvessel density [29]. Wang et al. reported that hASH1 activates Bcl-2 transcription, suggesting that Bcl-2 may be involved in hASH1-induced tumorigenic effects, so the blocking of hASH1 can potentially block Bcl-2 activity [51].

A reported case of recurrent ONB overexpressed both VEGF and KDR in the tumor tissue [28]. This study suggested that dysregulated VEGF/KDR signaling contributed to the tumor’s pathogenesis and progression through enhanced blood vessel formation. Sunitinib, a small-molecule inhibitor targeting multiple receptor tyrosine kinases, including KDR, was administered in combination with cetuximab (an EGFR-targeting monoclonal antibody) to this patient. This personalized treatment approach led to a marked reduction in the tumor size within five days and a complete radiologic response within one month, demonstrating the therapeutic value of targeting the VEGF/KDR axis in ONB [28].

Tumor necrosis factor-related apoptosis-inducing ligand (TRAIL) is a member of the tumor necrosis factor (TNF) superfamily. In a study by Koschny et al., the expression and functional activity of the TRAIL pathway were evaluated in primary human ONB cells [52]. Although these cells expressed key components of the TRAIL signaling pathway—including death receptors TRAIL-R1 and TRAIL-R2—they were found to be completely resistant to TRAIL-induced apoptosis [52]. Treatment with bortezomib, a proteasome inhibitor, at subtoxic concentrations sensitized the ONB cells to TRAIL, leading to significant apoptosis. Bortezomib enhanced the effectiveness of TRAIL by upregulating TRAIL-R2 expression on the tumor cell surface, enhancing TRAIL death-inducing signaling complex (DISC) formation, downregulating the antiapoptotic protein cFLIPL, and increasing the caspase-8/cFLIP ratio, thereby facilitating the activation of the apoptotic cascade [52]. The combinatorial treatment not only triggered apoptosis but also reduced the long-term clonogenic potential of ONB cells. Tumor cells that survived the initial treatment could still be resensitized by bortezomib, supporting the feasibility of repeated administration in clinical settings. The findings showed that, while ONB cells were intrinsically resistant to TRAIL monotherapy, TRAIL in combination with bortezomib holds significant promise as a targeted therapeutic strategy for this challenging tumor type [52].

## 4. Genomic Profiling of ONB

Recent analyses involving the multi-platform next-generation sequencing (NGS) of ONB have led to significant new insights into disease development. One investigation involved whole-genome sequencing (WGS) on metastatic ONB, revealing 62 short-nucleotide variants (SNVs) and five small insertions/deletions [53]. Notably, seven validated SNVs were detected in *MAP4K2*, *SIN3B*, *TAOK2*, *KDR*, *TP53*, *MYC*, and *NLRC4*, and the subsequent analysis of archived material indicated that *TP53*, *TAOK2*, and *MAP4K2* alterations were present in the primary tumor, whereas KDR, MYC, SIN3B, and NLRC4 arose only in metastatic lesions [53]. This observation suggests that ONB progression may entail early foundational lesions—such as *TP53* disruption—followed by additional “late” driver events under the pressure of therapy or other factors [53]. Building on this work, Cha et al. employed whole-exome sequencing (WES), whole-transcriptome sequencing, and OncoScan™ (Affymetrix, Santa Clara, CA, USA) copy number variation (CNV) testing to investigate seven rare metastatic adolescent and young adult cancers, including one ONB [54]. They discovered a *TP53* missense mutation and the loss of function of *CDKN2C* in ONB, positing that the inactivation of key tumor suppressors could be instrumental in ONB pathogenesis. From another perspective, based on the interactions of MDM-related pathways—specifically MDM-2—and *p53*, it may be promising in the development of treatment strategies in ONB management, as MDM-2 regulates *p53* activity, with the final result of an immediate p53-mediated response [55,56,57,58].

Furthermore, Helen et al. analyzed 18 formalin-fixed, paraffin-embedded primary and recurrent ONB cases using a combination of immunohistochemistry for *p53* and WAF-1 (a downstream target of *p53*), along with topographic genotyping to detect point mutations in the *p53* gene [43]. p53 immunohistochemical staining was weak to moderate in most cases, with some samples showing focal immunopositivity. No strong diffuse p53 immunostaining was observed, which is often associated with mutations in *p53* that result in its accumulation. The study found that no point mutations were detected in exons 5–8 of the *p53* gene in the ONB samples, suggesting that the observed immunopositivity was not due to the mutation-driven accumulation of the protein but rather the expression of wild-type *p53* [43]. WAF-1 expression was observed in cases showing p53 immunopositivity, supporting the hypothesis of *p53* wild-type hyperexpression. The study concluded that *p53* wild-type hyperexpression, characterized by the presence of both *p53* and WAF-1 immunopositivity, could serve as a marker for aggressive ONB. This hyperexpression was particularly prevalent in recurrent or metastatic tumors, suggesting that the presence of the wild-type *p53* protein may correlate with more aggressive tumor behavior and an increased likelihood of recurrence, although the study found no direct evidence of *p53* mutations driving tumor development [43].

Subsequent large-scale sequencing efforts highlight the wide genetic heterogeneity in ONB. A hybrid capture-based NGS approach applied to 41 relapsed or refractory ONBs found that 68% harbored at least one somatic genomic alteration, and 51% carried clinically relevant genomic alterations with therapeutic potential [59]. On average, 1.5 alterations were detected per tumor, with *TP53* mutations found in 17% of cases and additional potentially actionable changes involving *PIK3CA*, *NF1*, *CDKN2A*, and *CDKN2C* (7% each). Other, less frequent alterations included *PTCH1*, *CTNNB1*, *IDH2*, *ARID1A*, and *TSC1*, and 27% of tumors carried disruptions in the PI3K/mTOR pathway—including *PIK3CA*, *PTEN*, *RICTOR*, and *TSC1*—while 15% displayed alterations in genes associated with CDK-mediated cell cycle regulation (e.g., *CDKN2A*, *CDKN2C*, *CDK6*) [59].

In parallel, Topcagic et al. employed a multi-platform molecular profiling approach—encompassing next-generation and Sanger sequencing, whole-genome RNA microarrays, fluorescence in situ hybridization (FISH), IHC, and gene fusion analysis—on 23 formalin-fixed paraffin-embedded ONB samples to identify actionable genomic and proteomic alterations [26]. Mutational analysis revealed that 63% of ONBs harbored mutations, notably in *TP53*, *CTNNB1*, *EGFR*, *APC*, *cKIT*, *cMET*, *PDGFRA*, *CDH1*, *FH*, and *SMAD4*. Their study also noted the consistent overexpression of CD24, SCG2, and IGFBP-2, alongside the underexpression of ABCA8 and GHR, underscoring the complex genomic and transcriptomic landscape of ONB [26]. The most frequently altered pathway was Wnt/β-catenin, implicating *CTNNB1*, *APC*, and *CDH1*. Microarray data demonstrated significant transcriptional dysregulation (21 genes were consistently upregulated and 19 downregulated), while gene amplifications (e.g., *EGFR*, *HER2*, *cMET*) and gene fusions were absent. Novel upregulated oncogenic drivers included *CD24*, *SCG2*, and *IGFBP2*, while downregulated genes such as *ABCA8* and *GHR* may reflect suppressed transport and growth factor signaling in ONB. Moreover, 67% of cases expressed pan-NTRK proteins without underlying fusions, suggesting uncertain but potential relevance to NTRK-targeted therapies. Conversely, PD-L1 expression was absent in all cases, indicating the limited applicability of immune checkpoint inhibitors in this cohort.

Cracolici et al. evaluated somatostatin receptor 2 (SSTR2) expression in 78 ONB samples via IHC [60]. Although this study did not include extensive high-throughput sequencing, it revealed that 99% of ONBs strongly expressed SSTR2, regardless of the histologic grade or metastatic status [44]. Such universal SSTR2 positivity underscores the potential for somatostatin analog imaging (e.g., 68Ga-DOTATATE) and peptide receptor radionuclide therapy (PRRT) in advanced ONB, thereby complementing the growing list of genetically informed treatment possibilities.

Furthermore, a comprehensive molecular profiling study conducted by Lazo de la Vega et al. identified recurrent amplifications of the *FGFR3* gene in a significant subset of ONB cases [3]. High-level *FGFR3* amplification was observed in 5 out of 18 evaluable tumors (28%) in their analysis of 20 ONB tumors. RNA sequencing confirmed that *FGFR3* amplification was associated with markedly increased gene expression. *FGFR3* alterations in ONB primarily arose via copy number gains, which were sufficient to induce overexpression. ONB samples lacking *FGFR3* amplification showed significantly lower expression, reinforcing the biological relevance of amplification as a mechanism of activation and demonstrating the potential of *FGFR3* as an oncogenic driver [3]. *FGFR*-targeted therapies, such as multi-kinase inhibitors, may enable the guidance of targeted treatment modalities.

ONB also cytogenetically demonstrates high chromosomal instability, marked by frequent gains and losses across chromosomes. Guled et al. analyzed 13 ONB cases using oligonucleotide-based comparative genomic hybridization (CGH), revealing extensive and complex genomic CNVs, with a predominance of chromosomal gains over losses (8.37% vs. 7.36%) [61]. Several recurrent alterations were identified, including gains at *7q11.22–q21.11*, *13q*, *20q* and losses at *2q31.1–q37.1* and *6q16.3–q22.1*. High-stage (Kadish stage 3) tumors demonstrated a significantly higher burden of genomic instability, including whole-chromosome gains/losses and localized amplifications. For example, gains at *13q14.2–q14.3*, *13q31.1*, and *20q11.21–q11.23* and a loss at *Xp21.1* were observed in over 66% of high-stage tumors, suggesting their role in tumor progression. Furthermore, the study highlighted key candidate genes within these altered regions that may underlie ONB pathogenesis. Gains at 7q11.2 implicated LIMK1 and FZD9—genes associated with invasion and Wnt signaling—as potential oncogenic drivers. Amplifications at *20q13.32–q13.33*, a known hotspot in multiple carcinomas, may harbor the BRK tyrosine kinase gene (*PTK6*), linked to high-grade ovarian carcinomas. Conversely, deletions at *2q33.3* involving ADAM23, a neural adhesion-related tumor suppressor, were found in 50% of advanced cases, supporting its putative role in ONB malignancies. Other significant deletions included the *6q21–q22* region containing *FOXO3* and *CCNC*, both of which are implicated in tumor suppression and cell cycle regulation. This study suggested that the progression of ONB is accompanied by specific genomic alterations, particularly gains in 13q and 20q and losses in 2q and 6q, paralleling the changes seen in other aggressive epithelial malignancies.

Comparative genomic hybridization (CGH) and next-generation sequencing (NGS) studies have revealed associations between gains in *20q* and poor prognosis, analogous to findings in ovarian and breast cancers [62]. A CGH analysis by Valli et al. revealed gains in chromosomes 20, 14, 15, 5, 6, 7, 18, 19, and 22 as the most recurrent numerical changes, with gains being more common than losses [63]. However, no single chromosomal alteration was consistently recurrent across all cases. While some tumors had predominantly whole-chromosome gains, others displayed focal segmental rearrangements. One sample showed no detectable chromosomal imbalances. A comparison between a primary and relapsed tumor from the same patient revealed clonal evolution, with additional segmental changes in the relapsed lesion. Gay et al. observed gains in chromosome 5q regions (encompassing *FLT4*, *PDGFRB*, *FGFR4*, and *RICTOR*) [59]. Another subset of cases demonstrated non-focal amplifications in regions such as 20q and 8q, pointing to additional stratification potential within the molecular taxonomy of ONB.

## 5. Genomic Subclassification of ONB

Several recent studies have employed multi-omic approaches to subclassifying ONB, with potential impacts on prognosis and therapeutic approaches. Bell et al. identified “neural-like” and “basal-like” tumors, which are transcriptionally and epigenetically distinct molecular subtypes of ONB [12]. These subtypes reflect different ontogenetic origins within the olfactory mucosa, with neural-like ONBs hypothesized to arise from immature olfactory neuron progenitors, while basal-like ONBs may be derived from horizontal or multipotent GBCs. This distinction is supported by transcriptomic and DNA methylation profiling, where basal-like ONBs often exhibit distinct *IDH2 R172* mutations, a CpG island methylator phenotype (CIMP), and immune-enriched tumor microenvironments.

Classe et al. stratified ONBs into two biologically and clinically distinct subtypes: a “neural-like” and a “basal-like” group [27]. These subtypes were defined by distinct transcriptomic, proteomic, epigenomic, and immunologic characteristics that align with divergent cellular origins within the olfactory mucosa. The study identified frequent *IDH2 R172* mutations in approximately 35% of basal-like ONBs at the genomic level. *IDH2* mutations were associated with a CpG island methylator phenotype (E-CIMP), in line with the known impact of *IDH* mutations on epigenetics in other tumors. Chromatin remodeling genes, such as *ARID1A* and *SMARCA4*, were altered in 43% of cases. Basal ONBs demonstrated widespread hypermethylation, particularly in exon 1 regions, and the transcriptional repression of neuronal differentiation genes, implicating *IDH2* in a differentiation blockade. In addition to chromatin remodeling disruptions, alterations in DNA repair *(TP53*, *KMT2D*, and *NUMA1)* were also seen. The neural subtype exhibited enrichment in genes related to synaptic transmission and neural differentiation, including markers such as chromogranin A, synaptophysin, and S100-positive sustentacular cells. These tumors were well differentiated and associated with lower proliferation indices and favorable prognostic markers. The basal subtype showed the upregulation of genes involved in cell cycle regulation, basal cell carcinoma pathways, and embryonic development processes. These tumors presented as poorly differentiated, high-grade lesions with elevated Ki67 indices and increased necrosis. The basal subtype demonstrated transcriptional similarities with immature progenitor cells such as GBCs and immature neuronal progenitors (INP1), while neural ONBs were closer to more differentiated OSNs. Epigenomically, basal ONBs were hypermethylated at enhancer regions, while neural ONBs displayed hypomethylation at enhancer sites of axonal guidance genes, consistent with enhanced neuronal differentiation potential. Immunologically, basal ONBs exhibited significantly greater infiltration by CD4+ and CD8+ T cells and the elevated expression of immune checkpoint molecules (e.g., PD-1, PD-L1), cytotoxic markers (GZMB), and immunosuppressive mediators (IDO1, IL-10, FOXP3). Expectedly, basal tumors were associated with poorer outcomes, suggesting immune evasion mechanisms.

Capper et al. introduced the molecular stratification of ONB through genome-wide DNA methylation profiling, fundamentally challenging the histological diagnosis paradigm of this rare malignancy [64]. The study retrospectively analyzed 66 tumor samples, including ONB and other head and neck cancers, using DNA methylation arrays, IHC, CNV profiling, and the targeted NGS of 560 cancer-associated genes. This comprehensive multi-omics approach revealed substantial heterogeneity, leading to a refined classification into four distinct methylation-based subtypes. The four compared groups were core ONB, sinonasal tumors with *IDH2* mutations, sinonasal tumors with high methylation, and other sinonasal tumors. Core ONB (*n* = 42, 64%) was characterized by a classical lobular histoarchitecture, strong chromogranin A expression, the absence of or focal cytokeratin staining, and recurrent chromosomal losses (notably chromosomes 1–4, 8–10, and 12). NGS revealed rare recurrent mutations, with only *TP53* and *DNMT3A* mutated in 10% of samples each, underscoring the key driver mutations and relatively low overall mutation pattern of ONB. This subgroup aligns closely with the WHO-defined concept of ONB and represents the most homogeneous cluster in both DNA methylation and histopathology. The second group, sinonasal tumors with *IDH2* mutations (n = 7), displayed a distinct CIMP phenotype, histological features consistent with high-grade undifferentiated carcinoma, diffuse cytokeratin expression, and universal *IDH2 R172* hotspot mutations. These tumors closely resembled and clustered with previously reported *IDH2*-mutant sinonasal carcinomas, leading the authors to propose the sinonasal IDH2 carcinoma, a unified diagnostic term. This classification reflected significant molecular redefinition and aligned with the known IDH2-driven epigenetic reprogramming observed in other malignancies. The third minor group, sinonasal tumors with high methylation (n = 4), exhibited high global methylation but lacked *IDH1/2* mutations and specific defining molecular features. These tumors were distinguished by occurring in younger patients and displaying ambiguous IHC profiles, albeit histologically similar to ONB. Their rarity and undefined molecular identity suggested a possible novel or undercharacterized ONB-like tumor entity. Other sinonasal tumors (n = 13) displayed heterogeneous methylation profiles and IHC markers overlapping with sinonasal squamous cell carcinoma, adenocarcinoma, and undifferentiated carcinoma. These tumors were likely misdiagnosed ONBs due to their non-specific neuroendocrine features, reaffirming the diagnostic challenge posed by histology alone.

Batchu et al. conducted an integrative transcriptomic analysis to investigate tumor-infiltrating immune cell (TIIC) profiles across the basal and neural subtypes of ONB [65]. The study applied CIBERSORTx, a machine learning-based deconvolution tool, to assess immune cell infiltration and its relation to the tumor subtype and pathology, using RNA sequencing data from 18 ONB samples (nine basal and nine neural). Although the overall survival did not differ significantly between the basal and neural subtypes in this small series, the basal subtype displayed more aggressive pathological characteristics, including increased necrosis, an elevated Ki-67 proliferation index, and higher mitotic activity. Additionally, IDH2 mutations were uniquely present in the basal subtype. RNA deconvolution revealed subtle yet potentially significant differences in the immune composition between subtypes, despite the lack of statistical differences in gross IHC markers. M2 macrophages were identified as the most prevalent immune subpopulation across all samples, suggesting an immunosuppressive tumor microenvironment. Basal subtype tumors exhibited increased levels of activated CD4 memory T cells, while neural subtypes were characterized by a higher presence of resting dendritic cells. The findings indicate that, although principal component analysis could not clearly differentiate subtypes based solely on the immune cell proportions, specific immune subsets, such as CD4 memory T cells, may contribute to the more aggressive phenotype seen in basal ONBs. This study reinforces prior genomic classifications that distinguished ONB into basal and neural subtypes with divergent clinical behavior [27].

## 6. Current Management, Knowledge Gaps, and Implications for Future Management

With the advent of a combined multimodal approach for the treatment of ONB, outcomes for this tumor have dramatically improved since the original description of the tumor by Berger in 1924 [1]. However, due to the rarity of ONB, the standard-of-care treatment is not universally codified [66]. Recently, there has been a shift from subtotal and total resections to endoscopic transnasal/endonasal procedures with adjuvant RT or stereotactic radiosurgery [66]. The adjuvant use of radiation and chemotherapy has also shown some benefits [66].

Several gaps in understanding ONB remain due to the disease’s rarity, which impacts diagnosis, treatment, and long-term outcomes. While chemotherapy is used in advanced, unresectable, or recurrent ONB, its efficacy and optimal regimens remain unclear. Additionally, there are currently limited data on the best agents, combinations, or timing (neoadjuvant vs. adjuvant chemotherapy) of chemotherapy treatment. Recent advances in radiotherapy have provided new radiation techniques that show a great deal of promise in improving OS and PFS; however, due to the rarity of ONB, there is limited evidence of their long-term toxicity, especially in terms of preserving brain and optic nerve function. Although there has been a trend toward a multimodal approach to ONB treatment, the interaction and utilization of surgery, radiation, and chemotherapy are not standardized. Thus, it is still unclear exactly how to tailor multimodal therapy to different stages or grades of ONB. In terms of the molecular pathways and targeted therapies toward them, ONB shows the involvement of pathways like EGFR, PI3K/AKT, MAPK/ERK, and HIF-1a/Epo/EpoR/Bcl-2 and differentiation by distinct genomic subclasses. Tailoring treatments to target these pathways would prove beneficial for personalized treatment.

A case report by Wang et al. demonstrated a successful treatment with a combination of cetuximab, a targeted drug for *EGFR*, and sunitinib in a patient with recurrent ONB [28]. This study was the first to demonstrate the successful treatment of ONB with targeted drugs [28]. Another case reported by Preusser et al. demonstrated a successful disease stabilization procedure with sunitinib mesylate in a patient with recurrent ONB [67]. Sunitinib mesylate is an oral multityrosine kinase inhibitor that targets receptors like c-kit, FLT3, KDR, PDGFR, RET, and VEGFR [68]. Immunohistochemical analysis of the tumor tissue revealed the notable expression of PDGFR-b on stromal and endothelial cells, but c-kit expression was not significant in tumor cells. The authors hypothesized that the inhibition of PDGFR-b contributed to the favorable clinical outcome, although further studies are needed to validate this finding and to understand the full therapeutic potential of sunitinib mesylate and the role of its targets, including c-kit, in such tumors. Furthermore, Kim et al. used imatinib mesylate as a second-line treatment in an EGFR-negative and c-kit-positive ONB case and emphasized the need for further investigation into this issue [69].

Gay et al. provided rational grounds for the application of targeted therapeutics, including tyrosine kinase inhibitors like sunitinib and everolimus [59]. Stable disease was observed in patients harboring alterations in *PTCH1*, *PIK3R2*, and *KIT* when treated with vismodegib, everolimus, or sunitinib, respectively. The study, being one of the most comprehensive genomic analyses of ONB to date, did not only validate the potential of CGP in unveiling actionable alterations but also suggested the feasibility of the molecular classification of ONB subtypes based on mutational signatures and pathway dependencies. Integrating CGP into routine diagnostic workflows may significantly impact the management of recurrent or refractory ONB as the availability of targeted therapies expands.

Altogether, the collective evidence from these studies emphasizes that ONB arises through a continuum of early and late oncogenic events, which may be further shaped by therapy or other selective pressures. Through the evolving lens of genomic and immunohistochemical analyses, researchers and clinicians are progressively identifying new diagnostic markers and therapeutic targets, offering a tangible path toward precision treatment strategies for this rare and challenging malignancy. The future management of ONB will focus on improving early detection, refining treatment strategies, and integrating novel therapies to enhance patient outcomes. Advances in molecular profiling may help to identify biomarkers for prognosis and targeted therapies, leading to more personalized treatment approaches. Surgical management is expected to continue evolving, with minimally invasive endoscopic and robotic-assisted techniques reducing morbidity while maintaining oncologic control. Radiation therapy will likely see further advancements in conjunction with targeted therapies. Additionally, emerging immunotherapy and targeted therapies may provide new treatment options, particularly for recurrent or advanced cases. Optimizing multimodal treatment approaches by refining the sequence and combination of surgery, radiation, and chemotherapy could improve long-term disease control. Improved imaging techniques will work to enhance surveillance and may enable the earlier detection of recurrence, allowing for timely intervention. Finally, ongoing clinical trials will be crucial in evaluating the efficacy of new drugs, immunotherapies, and combination strategies, potentially reshaping the standard of care for ONB and improving the survival rates while enhancing the quality of life of patients.

## 7. Conclusions

ONB is a rare and biologically complex neuroectodermal malignancy, characterized by a heterogeneous clinical frame and diverse molecular features. The integration of multi-omics approaches with the help of recent advances in genomic, transcriptomic, and epigenomic analyses has provided novel insights into the molecular background of ONB driving tumor progression and therapeutic resistance, implicating neural crest-derived oncogenesis and revealing the dysregulation of critical signaling pathways, immune microenvironment modulation, and dynamic tumor–stroma interactions. NGS has helped to identify novel diagnostic and prognostic markers, including NEUROD1, OTX2, and SSTR2. Moreover, the delineation of the immunosuppressive tumor microenvironment, characterized by T cell exclusion, myeloid-derived suppressor cell (MDSC) infiltration, NK cell paucity, and MHC downregulation, offers potential for the future application of immunotherapeutic interventions.

Despite these promising molecular advances, translation into clinical practice remains limited due to the rarity of ONB, the absence of standardized molecular-driven treatment algorithms, and the scarcity of large, prospective clinical trials. Future efforts should prioritize the systematic integration of multi-omics data into clinical management and focus on multi-institutional collaboration to validate biomarkers, elucidate therapeutic vulnerabilities, and optimize precision oncology strategies. The continued expansion of the molecular understanding of ONB will be crucial in facilitating earlier diagnosis, enhancing personalized therapeutic strategies, overcoming the current therapeutic limitations, and improving the long-term outcomes for patients afflicted with this rare and challenging malignancy.

## Figures and Tables

**Table 1:** Molecular alterations in ONB.

Reference	Gene Alteration	Study Type	Function in ONB
[23]	*ATRX*	Human and cell lines	Chromatin remodeling protein; maintains telomere stability; ATRX loss leads to genomic instability, alternative lengthening of telomeres, and aggressive tumor behavior
[24]	*BCL-2*	Human	Antiapoptotic protein; elevated expression in ONB; promotes angiogenesis in malignant tumors
[25]	*BMP7*	Human	Member of the bone morphogenetic protein family involved in mesenchymal differentiation and extracellular matrix remodeling, characteristic of the mesenchymal ONB subtype
[26,27]	*CDKN2A*	Human	Encodes tumor suppressors p16INK4a and p14ARF; loss of *CDKN2A* leads to uncontrolled cellular proliferation
[26]	*CTNNB1*	Human	Gene encodes beta-catenin; mutations result in constitutive activation of Wnt signaling and lead to enhanced cellular proliferation, reduced apoptosis, and increased oncogenic potential
[28]	*EGFR*	Human	May be activated via TRKB; promotes cell proliferation and inhibits cell apoptosis
[29]	EPO, EPOR	Human	Molecular marker present in ONB cells; binding with EPOR promotes angiogenesis
[30]	*ERK*	Human	Promotes transcription of factors that drive cell proliferation and prevent apoptosis; activated by TRKB
[7,31]	*EZH2*	Integrated human–mouse single-cell atlas—human	Stemness marker; potential therapeutic target; silences tumor suppressor genes through trimethylation of histone H3 at lysine 27 (H3K27me3) as a histone methyltransferase
[28]	*FGFR2*	Human	Receptor tyrosine kinase; drives tumor growth through activation of downstream signaling pathways such as MAPK and PI3K/AKT; alterations suggest potential for targeted therapy in ONB
[32]	*GRP78*	Human	Chaperone protein; regulates the unfolded protein response, protecting cells from stress-induced apoptosis; overexpression indicates a role in tumor adaptation to hypoxic and metabolic stress conditions and promotes survival and therapeutic resistance
[17]	*hASH-1*	Human	Encoded by *ASCL1* gene; overexpression may act as a trigger for cancer formation in olfactory epithelial cells; involved in lineage specification, neuronal commitment, and differentiation; downregulated via Notch pathway; activates *BCL-2*
[7]	HES6	Integrated human–mouse single-cell atlas	Transcription factor; regulates neuronal differentiation; represses Notch signaling, promoting cell cycle exit and differentiation
[29]	HIF-1a/EPO/EPOR/Bcl-2	Human	Leads to autocrine signaling, which promotes angiogenesis through Bcl-2
[29]	HIF1A	Human	Transcription induced by phosphorylated STAT3; induces EPO and EPOR expression in ONB cells
[27,31]	*IDH2* mutations	Human-Human	Identified in a subset of ONB cases with atypical epithelial differentiation, often associated with more aggressive behavior; leads to the production of the oncometabolite 2-hydroxyglutarate (2-HG), which inhibits α-KG-dependent dioxygenases, including histone demethylases and ten-eleven translocation (TET) family DNA demethylases; results in DNA hypermethylation, epigenetic reprogramming, and cellular differentiation blockade
[33]	*LAMA2*	Human	Regulates extracellular matrix integrity and cell adhesion; potential role in tumor invasion
[30]	*MAPK/ERK*	Human	Enhances the maintenance of brain tumor-initiating cells (BTICs); prevents apoptosis and increases cell proliferation; promotes lung adenocarcinoma metastasis formation through expression of TRKB
[34]	*NEUROD1*	Human	Transcription factor; enhances neuronal maturation by activating genes involved in synapse formation; plays a role in neural lineage commitment and synaptic development; helps in distinguishing ONB from SNUC
[30]	*PI3K/AKT*	Human	Activated via overexpression of TRKB; prevents apoptosis, causes an increase in cell growth, and promotes angiogenesis
[35]	*OTX2*	Human	Homeobox gene; plays a critical role in embryonic brain and neural crest development; overexpression promotes tumor cell proliferation by activating neurodevelopmental pathways and leads to enhanced oncogenesis
[28]	*RET*	Human	Receptor tyrosine kinase involved in cell survival and proliferation; *RET* mutations have been implicated in ONB oncogenesis, and targeted therapies against RET are currently under investigation
[5]	Rb1 protein	Animal	Tumor suppressor protein that controls the G1/S transition of the cell cycle; binds to and inhibits E2F transcription factors, preventing excessive cell cycle progression when it is functional; loss of function leads to dysregulated cell division and is associated with neuroendocrine differentiation
[36]	*SETD2*	Human	Histone methyltransferase; regulates gene expression by modifying chromatin structure; catalyzes trimethylation of histone H3 at lysine 36 (H3K36me3); mutations impair transcriptional regulation, DNA repair, and chromatin integrity
[30]	*STAT3*	Human	Activated by phosphorylation in ONB; triggers increased transcription of HIF1A
[37,38,39,40]	*SMARCB1*	Human	Critical component of the SWI/SNF chromatin remodeling complex; regulates gene expression by altering nucleosome positioning; mutations or deletions lead to epigenetic dysregulation, loss of differentiation, and increased tumorigenicity
[41,42]	*SMARCA4*	Human	SWI/SNF component; encodes the ATPase Brg1, which is essential for chromatin modeling; loss-of-function mutations lead to transcriptional deregulation and are associated with highly aggressive ONB subtypes
[43]	*TP53* mutations	Human	Implicated particularly in high-grade cases of ONB and correlates with poor prognosis; results in uncontrolled cell proliferation, increased genomic instability, and resistance to apoptosis; may confer susceptibility to WEE kinase inhibitors, which regulate the DNA damage response, providing a promising therapeutic approach
[32]	TrkA	Human	Strongly expressed in ONB; high-affinity neurotrophin receptor; not expressed in brain tumor-initiating cells; participates through the *PI3/AKT* pathway; promotes proapoptotic and antiangiogenic effects
[32]	TrkB	Human	Strongly expressed in ONB; high-affinity neurotrophin receptor; binds BDNF; participates in the *MAPK/ERK* and *PI3/Akt* pathways; induces tumorigenesis; enhances maintenance of brain tumor-initiating cells; promotes lung adenocarcinoma metastasis formation

BCL-2: B-cell lymphoma-2; EPO: erythropoietin; EPOR: erythropoietin receptor; *ERK*: extracellular signal-regulated kinase; *hASH-1*: human achaete-scute homolog 1 (*hASH1*); *ASCL1*: achaete-scute homolog-1; HIF1A: hypoxia-inducible factor 1 subunit alpha; STAT3: signal transducer and activator of transcription 3; BDNF: brain-derived neurotrophic factor; TrkA: tropomyosin receptor kinase A; TrkB: tropomyosin receptor kinase; IDH2: isocitrate dehydrogenase; EZH2: enhancer of zeste 2 polycomb repressive complex 2 subunit; *TP53*: tumor protein 53; *NEUROD1*: neuronal differentiation; Rb1: retinoblastoma 1; *SMARCB1*: SWI/SNF-related, matrix-associated, actin-dependent regulator of chromatin subfamily B, member 1; *SMARCA4*: SWI/SNF-related, matrix-associated, actin-dependent regulator of chromatin, subfamily A, member 4; ATRX: alpha thalassemia/mental retardation syndrome X-linked; SETD2: SET domain-containing 2; CDKN2A: cyclin-dependent kinase inhibitor 2A; RET: rearranged during transfection; FGFR2: fibroblast growth factor receptor 2; *CTNNB1*: catenin beta 1; GRP78: glucose-regulating protein 78; HES6: Hes family BHLH transcription factor 6; BMP7: bone morphogenetic protein 7; LAMA2: laminin subunit alpha-2; *EGFR*: epidermal growth factor receptor; MAPK/ERK: mitogen-activated protein kinase/extracellular signal-regulated kinase; PI3K/AKT: phosphatidylinositol 3-kinase/protein kinase B.

## Data Availability

Not applicable.

## Abbreviations

The following abbreviations are used in this manuscript:

MDSC	Myeloid-derived suppressor cells
NK	Natural killer
ONB	Olfactory neuroblastoma
TAM	Tumor-associated macrophage
TME	Tumor microenvironment
